# Computationally identified novel agonists for GPRC6A

**DOI:** 10.1371/journal.pone.0195980

**Published:** 2018-04-23

**Authors:** Min Pi, Karan Kapoor, Ruisong Ye, Dong-Jin Hwang, Duane D. Miller, Jeremy C. Smith, Jerome Baudry, L. Darryl Quarles

**Affiliations:** 1 Department of Medicine, University of Tennessee Health Science Center, Tennessee, United States of America; 2 UT/ORNL Center for Molecular Biophysics, Oak Ridge, Tennessee, United States of America; 3 Department of Pharmaceutical Sciences, University of Tennessee Health Science Center, Memphis, Tennessee, United States of America; 4 Department of Biochemistry and Cellular and Molecular Biology, University of Tennessee, Knoxville, Tennessee, United States of America; Danish Cancer Society Research Center, DENMARK

## Abstract

New insights into G protein coupled receptor regulation of glucose metabolism by β-cells, skeletal muscle and liver hepatocytes identify GPRC6A as a potential therapeutic target for treating type 2 diabetes mellitus (T2D). Activating GPRC6A with a small molecule drug represents a potential paradigm-shifting opportunity to make significant strides in regulating glucose homeostasis by simultaneously correcting multiple metabolic derangements that underlie T2D, including abnormalities in β-cell proliferation and insulin secretion and peripheral insulin resistance. Using a computational, structure-based high-throughput screening approach, we identified novel tri-phenyl compounds predicted to bind to the venus fly trap (VFT) and 7-transmembrane (7-TM) domains of GPRC6A. Experimental testing found that these compounds dose-dependently stimulated GPRC6A signaling in a heterologous cell expression system. Additional chemical modifications and functional analysis identified one tri-phenyl lead compound, DJ-V-159 that demonstrated the greatest potency in stimulating insulin secretion in β-cells and lowering serum glucose in wild-type mice. Collectively, these studies show that GPRC6A is a “druggable” target for developing chemical probes to treat T2DM.

## Introduction

The prevalence of MetS is increasing in parallel with rising prevalence of T2DM, and contributes significantly to morbidity and mortality globally [1]. The multitude of metabolic derangements present in MetS and T2DM create a significant challenge to treatment efforts. The chronic β-cell decompensation, owing to impaired glucose-sensing and insufficient increases in β-cell mass [2], peripheral insulin resistance, and impaired suppression of hepatic glucose production, as well as other factors coalesce over time to cause overt T2D. Lowering of blood glucose remains the priority in treating T2DM. To-date, monotherapies for glycemic control in T2D typically target only one of these multiple derangements, and therefore are typically used in combination. There remains an unmet need to identify new therapeutic targets to improve glycemic control.

GPRC6A, a family C G protein coupled receptor, is proposed to be master regulator of energy metabolism [3, 4]. This receptor is expressed in key metabolic tissues and plays a central role in regulating organ-specific functions controlling systemic glucose and fat metabolism, including direct actions in pancreatic β-cells, liver hepatocytes, and skeletal muscle. In mice, GPRC6A also controls inter-organ communications through the coordinated secretion of insulin from β-cells, GLP-1 from intestinal cells, testosterone (T) from Leydig cells, IL-6 from myocytes. Thus, targeting GPRC6A activation represents a potential paradigm shifting opportunity to make significant strides in treating and preventing T2DM by simultaneously targeting abnormalities in β-cells, hepatocytes and skeletal muscle as well as stimulating an ensemble of metabolically active hormones [5].

GPRC6A is unusual in that it is activated by multiple endogenous ligands, including osteocalcin (Ocn), T, basic amino acids, such as L-Arginine, and cations, such as calcium. GPRC6A is able to sense dissimilar ligands because of its unique structure that has two distinct binding domains, namely a periplasmic nutrient venus fly trap (VFT) motif that is fused to a classical heptahelical 7 transmembravfne (7-TM) domain. Distinct ligand binding sites in the VFT and 7-TM domains are purported to provide the structural basis for both independent biological and pharmacological actions of orthosteric ligands and allosteric modulators with different affinities and efficacies.

Genetic and pharmacological studies have validated the importance of GPRC6A in regulating energy metabolism. Ablation of *Gprc6a* in mice results in obesity, glucose intolerance, hepatic steatosis, sarcopenia and insulin resistance [6], and deletion of Ocn, a natural ligand for GPRC6A leads to an identical phenotype to *Gprc6a*^*-/-*^ mice [7]. Administration of Ocn also improves glucose tolerance, increases insulin sensitivity, β-cell mass and insulin secretion [8, 9], reduces fat, increases muscle mass and reverses hepatosteatosis in mice feed high fat diets. Ocn also stimulates GLP-1 and testosterone (T) secretion through GPRC6A in intestinal cells [10–12] and Leydig cells [13–16], respectively. Genetically modified mice with an increase expression of uncarboxylated Ocn are protected from T2DM and obesity [8]. Clinically, Ocn and T improve insulin secretion, and insulin sensitivity in models of T2D, and T is associated with reduced mortality in T2D [17–20].

There is an unmet need to develop drugs that bind to and activate GPRC6A. The use of naturally occurring ligands for GPRC6A to treat T2D is limited by the fact that Ocn is a peptide that requires systemic administration in large concentrations. Indeed, Ocn treatment at the doses of 1 and 3 μg/kg are required to stimulate insulin levels and insulin gene expression, respectively, in mice. Other natural ligands, such as L-arginine also require doses in excess of 2 g/kg and the response is transient. L-arginine and T also have other metabolic effects that limit their utility as treatments for T2D.

In previous studies, we developed computational structural models of the Venus Fly Trap (VTF) and 7-transmembrane (7-TM) domain as a means to examine the structural basis for ligand binding to GPRC6A [21] [22]. Docking studies, confirmed by site-directed mutagenesis, have identified the residues that contribute to T [21] and Ocn [22] binding in the 7-TM of GPRC6A. Calculations suggest that L-amino acids and gallic acid (GA) bind to the VFT, whereas Epigallocatechin 3-gallate (EGCG) is calculated to bind to sites in both the VFT and 7-TM [23]. In the current study, we used computational structural models for the VFT and 7-TM to create a consensus homology model of GPRC6A to perform virtual high throughput screening of chemical libraries to identify novel agonist chemical compounds for GPRC6A. We report on the resulting discovery of novel tri-phenyl scaffold-based compounds that activate GPRC6A *in vitro* and reduce serum glucose when administered to mice *in vivo*.

## Materials and methods

### Reagents

Curcumin, Gallic acid, Sodium gallate, Ethyl gallate, Octyl gallate, Catechin, Catechin gallate, Epigallocatechin, Epigallocatechin gallate, Metformin, Testosterone, L-Arginine and insulin were purchased from Sigma.

Osteocalcin was purified from bovine tibial bone extracts [24]. Briefly, cortical bone was obtained from the central section of the tibia of freshly slaughtered bovine from Piggly Wiggly (Memphis, TN). Bone samples were freed of marrow and connective tissue, ground to a particle size that passed through a 210-μm sieve, and washed with several changes of water for 24 hr at 4 °C. The bone was then dialyzed against several changes of 0.5 M EDTA, pH 8, at 4 °C for 8–10 days. The soluble fraction inside the dialysis sack was collected by centrifugation, dialyzed exhaustively against 5 mM NH4HCO3, and lyophilized. After gel filtration on Sephadex G-100, peak fractions containing the osteocalcin were lyophilized and then chromatographed on a 2 X 50 cm column of DEAE-Sephadex A25 at 25 °C with a linear gradient in 0.1 M Tris-HCI, pH 8.0, from 0 to 0.75 M NaCl. Decarboxylated osteocalcin was produced by treating osteocalcin *in vacuo* at 110 °C [25]. The purity and decarboxylation state were confirmed by native gel electrophoresis, or by blotting followed by reaction with DBS staining for γ-carboxyglutamic acid [26].

### Cell culture

All culture reagents were from Invitrogen. Human embryonic kidney HEK-293 cells were obtained from American Type Culture Collection. The mouse pancreatic β-cell, MIN-6 was purchased from AddexBio. HEK-293 cells stably transfected with pcDNA3.mGPRC6A were created as previously described [27, 28]. HEK-293 and HEK-293 transfected with a mouse GPRC6A cDNA cells [28, 29] were cultured in DMEM medium supplemented with 10% fetal bovine serum and 1% Penicillin/Streptomycin (P/S).

### Measurement of total and Phospho-ERK by Western-Blot analysis

Briefly, HEK-293 cells transfected with/without expressing GPRC6A was made quiescent by overnight incubation in serum-free DMEM/F12 containing 0.1% bovine serum albumin (BSA) and stimulated with various ligands at different doses. ERK activation was assessed 5–30 min after treatment by immunoblotting using anti-phospho-ERK1/2 MAP kinase antibody (Cell Signaling Technology) corrected for the amount of ERK using an anti-ERK1/2 MAP Kinase antibody (Cell Signaling Technology) to measure ERK levels.

### Insulin secretion assay

The MIN-6 cells were plated onto 24-well plates at a density of approximately 0.5X10^6^ cells/well and were grown to 90 ~ 100% confluence before assay. At 18 hours before secretion experiments, the standard tissue culture medium containing 11.1 mmol/L glucose was switched to fresh medium containing 5 mmol/L glucose. Insulin secretion was assayed in HEPES-balanced salt solution [114 mmol/L NaCl, 4.7 mmol/L KCl, 1.2 mmol/L KH2PO4, 1.16 mmol/L MgSO4, 20 mmol/L HEPES, 2.5 mmol/L CaCl2, 25.5 mmol/L NaHCO3, and 0.2% bovine serum albumin (essentially fatty acid free); pH 7.2] [30]. The insulin stimulation index was calculated as the ratio of stimulation media insulin concentrations in Ocn divided by the insulin concentration in control stimulation media (without GPRC6A agonists) at low glucose conditions [9, 31].

### Computational modeling and docking

The computational identification of candidate molecules was performed using an approach based upon the three-dimensional structure and dynamics of the target. To do this first models were constructed of the target protein TM and VFT domains, based on sequence homology with proteins of experimentally-determined 3D structure [21–23]. Next, the models were subjected to molecular dynamics (MD simulation to generate a description of the internal motions. Configurations were clustered and the resulting ensemble subjected to VHTS docking, in an approach known as ‘ensemble docking’; this approach has been successful in previous lead compound searches for a variety of targets [32].

### GPRC6A Venus-Flytrap (VFT) domain homology modeling

Structural models of the GPRC6A transmembrane [33] domain were developed using GluR-1 and GluR-5 templates as previously described [21, 22]. Venus Flytrap (VFT) domain modeling was performed as follows: multiple sequence alignment (MSA) of the GPRC6A sequence with eight family C GPCR sequences was performed using Fast Fourier Transform and E-INS-i methods [34]. The following family C sequences were used: 1) human metabotropic glutamate receptor (mGluR-1) (PDB code 4OR2), 2) human mGluR-5 (PDB code 4OO9), 3) mouse mGluR-3 (PDB code 2E4U), 4) human CasR, 5) human γ-aminobutyric acid B receptor-1 (GABAR-1) (PDB code 4MQE), 6) human probable GPCR-158, 7) human retinoic acid-induced protein-3, and 8) human taste receptor type-1 member-1. Sequences 4 and 6 through 8 have no available crystal structures, whereas sequences 1 and 2 have crystal structures for their transmembrane [33] domains, and sequences 3 and 5 for their extracellular domains. In addition, in the multiple alignment using Fast Fourier Transform MSA, seven crystal structures of the short extracellular domain, TM domain, and short cytoplasmic domain of family A sequences were also included. These sequences were: 1) bovine rhodopsin receptor (PDB code 3CAP), 2) turkey β-1 adrenergic receptor (PDB code 2VT4), 3) human β-2 adrenergic receptor (PDB code 2RH1), 4) human adenosine A2A receptor (PDB code 2YDV), 5) human 5-hydroxytryptamine receptor-2B (PDB code 4IB4), 6) human 5-hydroxytryptamine receptor-1B (PDB code 4IAR), and 7) rat neurotensin receptor type-1 (PDB code 4GRV).

The structure of the mGluR-3 (crystal structure of VFT domain) with the highest sequence similarity to GPRC6A was selected as the template for the VFT domain modeling. A missing region (residues 425–426) of the mGluR-3 template structure was modeled using GABAR-1 VFT structure. Ten main chain models with ten side chain conformers per main chain model were generated for the template using the MOE-2013 (Molecular Operating Environment, 2013.08; Chemical Computing Group, Inc) homology facility and the CHARMM27 force-field [35] implemented within MOE. The best-scoring homology model based on Coulomb and Generalized Born interaction energies (GB/VI) scores [36] was selected for further MD and docking studies.

### Ligand binding sites in GPRC6A models

Allosteric binding site residues in the TM domain have been identified and described previously [21]. The binding site residues for glutamic acid observed in the crystal structure of mGluR-3 were used to define the putative binding-site in the VFT domain model using MOE’s Site Finder facility. Docking of ligands to the VFT domain was carried out using the Docking facility in MOE with the CHARMM27 force-field. Binding site residue side chains were allowed to be flexible during the docking using a tethering weight of 0.1 kcal/mol·Å^2^. GB/VI free energy scores, as implemented in MOE, were used to rank poses of the docked ligands.

### Molecular dynamics simulations of GPRC6A TM region

The CHARMM-GUI [37] was used to build a molecular dynamics (MD) simulation system from the highest scoring energy-minimized homology models of the TM domain (sequence 582–844), generated from each of the two template structures of mGluR-1 and mGluR-5. A disulfide bond was built between Cys659 and Cys744, known to form a S-S bond in family C GPCRs. A membrane was built using layers of 1-palmitoyl-2-oleoyl-sn-glycero-3-phosphocholine (POPC) molecules around the protein, providing a homogeneous distribution of lipid molecules around the protein, and by hydrating the membrane:protein system with TIP3P water molecules 15Å above and below the protein. Five negative ions were added to neutralize the system and placed using the Monte-Carlo Placing method. The final system contained 43497 atoms in the case of the GluR-1 model and 45621 atoms in the case of the Glu-5 model. Periodic Boundary Conditions were applied. A switching distance of 1nm, cut-off value of 1.2nm and pair-list distance of 1.4nm were used to calculate van der Waals and electrostatic nonbonded interactions. A time-step of 2 fs/step was used for all calculations. A constant 300K temperature was maintained throughout the simulation using Langevin dynamics, with a damping coefficient of 1 ps^-1^. Particle Mesh Ewald (PME) was used for the calculation of the electrostatic interactions. The trajectory coordinates were written to disk every 2 ps.

The MD simulation was performed using NAMD-2.9 (MPI version) [38]. All simulations used CHARMM-36 parameters for protein, lipids, ions and water molecules [35]. Three equilibration steps were performed, as follows: 1) the lipid tails were equilibrated keeping the lipid head groups, water molecules and protein fixed. A 1000 steps conjugate gradient energy minimization was performed and a 0.5 ns NVT ensemble equilibration performed. 2) the protein was kept fixed and the system energy minimized for 2000 steps and equilibrated for 0.5 ns in the NPT ensemble using a Langevin piston Nose-Hoover method for maintaining a 1 bar pressure, using barostat oscillation and damping time scales of 200 fs and 50 fs, respectively. This allowed the area in the xy-plane to fluctuate and permit packing of lipids against the protein. 3) all restraints were removed and the system was energy minimized for another 2000 steps and then allowed to equilibrate for 1 ns in the NPT ensemble. Finally, a 100ns production trajectory was obtained. Structures of the protein along the MD trajectory were parsed for every 50 ps using Prody v1.2 [39], generating a total of 6000 structures. Pairwise average Linkage hierarchical clustering RMSD clustering was used to cluster these structures with Maxcluster [40] using the RMSD distance between the data point represented by the allosteric binding site residues identified previously. Potential binding pockets were identified using FTMAP [41] and a consensus binding site was built around the allosteric binding site residues in the TM domain.

### Molecular dynamics simulations of GPRC6A VFT region

The CHARMM-GUI was again used to build an MD simulation system for the highest scoring energy-minimized homology model of the VFT domain (sequence 27–516), generated using the template structure of mGluR-3. A rectangular TIP3P water-box type was built using a water thickness of 10Å around the protein. Four positive ions were added to neutralize the system and located using the Monte-Carlo Placing method. The final system contained 84178 atoms.

Periodic boundary conditions, the switching distance, cut-off distance, pair-list distance, temperature, PME and time-step were set similar to the GPRC6A TM system above and the simulations were performed in the NPT ensemble using Langevin dynamics. The MD simulation was generated using NAMD with two equilibrium steps performed as follows- 1) the protein was kept fixed and the water box was energy minimized for 1000 steps using the conjugate gradient method and equilibrated for 0.5 ns. 2) the restraints were removed and the system energy minimized for another 2000 steps and then allowed to equilibrate for 0.5 ns. Finally, a 100ns production trajectory was generated. The clustering of the trajectory and identification of binding pockets around the orthosteric binding site residues was carried out as in the TM domain analysis described above.

### High-throughput dual-docking approach

A docking approach targeting the two domains of the receptor by small molecules was used here. The goal of this approach was to identify molecules that bind well to either or both of the two domains, an approach designed to optimize the hit-rate i.e., the identification of suitable agonists/antagonists for the receptor. Virtual screening was performed using the program Autodock Vina [42] on 18 structures: 12 structures obtained from the TM MD clustering and 6 from the VFT MD clustering. The compound data bases used for docking were as follows: i) zinc annotated NCI Plated 2007, ii) zinc annotated NCI diversity set, iii) known allosteric TM agonists/antagonists of GluR-1, GluR-3, GluR-5, CasR and mouse-GPRC6A, iv) known orthosteric VFT agonists/antagonists of GluR-1, GluR-3, GluR-5, CasR and mouse-GPRC6A, and v) GPRC6A agonists/antagonists already tested by our group. The redundancies between the different subset of compounds were removed. This gave a total set of 89,350 compounds docked in both TM/VFT domains using Vina. The compounds were docked to each of the 18 structures from the MD simulations (6 each from the two TM models and 6 from the VFT model). Default values were used for the maximum number of binding modes to be generated. The value of the exhaustiveness parameter in Autodock Vina, which directly determines the extent of sampling of chemical space in the binding site, was set to 20.

### Prioritizing compounds for experimental testing

The computational hits were analyzed using two criteria- i) binding energies as predicted by Vina and ii) “snapshot count” [43], giving the number of MD snapshots to which one specific compound is predicted to bind. This allowed the identification of compounds predicted to bind with high binding affinities to multiple conformations of the TM and VFT domains of GPRC6A.

Two lists of compounds were prepared for each of the two domains for experimental validations: the first list containing compounds with highest binding affinities, and the second list containing compounds ranked in top-5000 (based on binding affinities) that can bind to maximum number of protein conformations (snapshots). The first list identifies compounds that are predicted to bind to the targets with a high binding score (approximation of binding affinity), even on rarely-sampled conformations, and the second list identifies compounds that are predicted to bind to conformations of the target that are sampled often.

### Blood glucose measurement

8 to 10 weeks ago wild type C57BL/6 mice were fasted for 5 hours, injected ip with DJ-V-159 (10 mg/kg body weight), or Metformin (300 mg/kg body weight), or vehicle (95% PEG + 5% DMSO; 10 μl/g body weight). Blood glucose levels were measured at 0, 30, 60 and 90 minutes after injection using blood glucose strips and the Accu-Check glucometer as described [44, 45].

Mice were maintained and used in accordance with recommendations as described (National Research Council 1985; Guide for the Care and Use of Laboratory Animals Department of Health and Human Services Publication NIH 86–23, Institute on Laboratory Animal Resources, Rockville, MD) and following guidelines established by the University of Tennessee Health Science Center Institutional Animal Care and Use Committee. The animal study protocol was approved by the institutional review boards at University of Tennessee Health Science Center Institutional Animal Care and Use Committee.

### Statistics

We evaluated differences between groups by one-way ANOVA, followed by a post hoc Tukey’s test. Significance was set at *P* < 0.05. All values are expressed as means ± SEM. All computations were performed using the Statgraphic statistical graphics system (STSC, Inc).

## Results

### Homology modeling and identification of binding sites for amino acids

**Fig 1:**
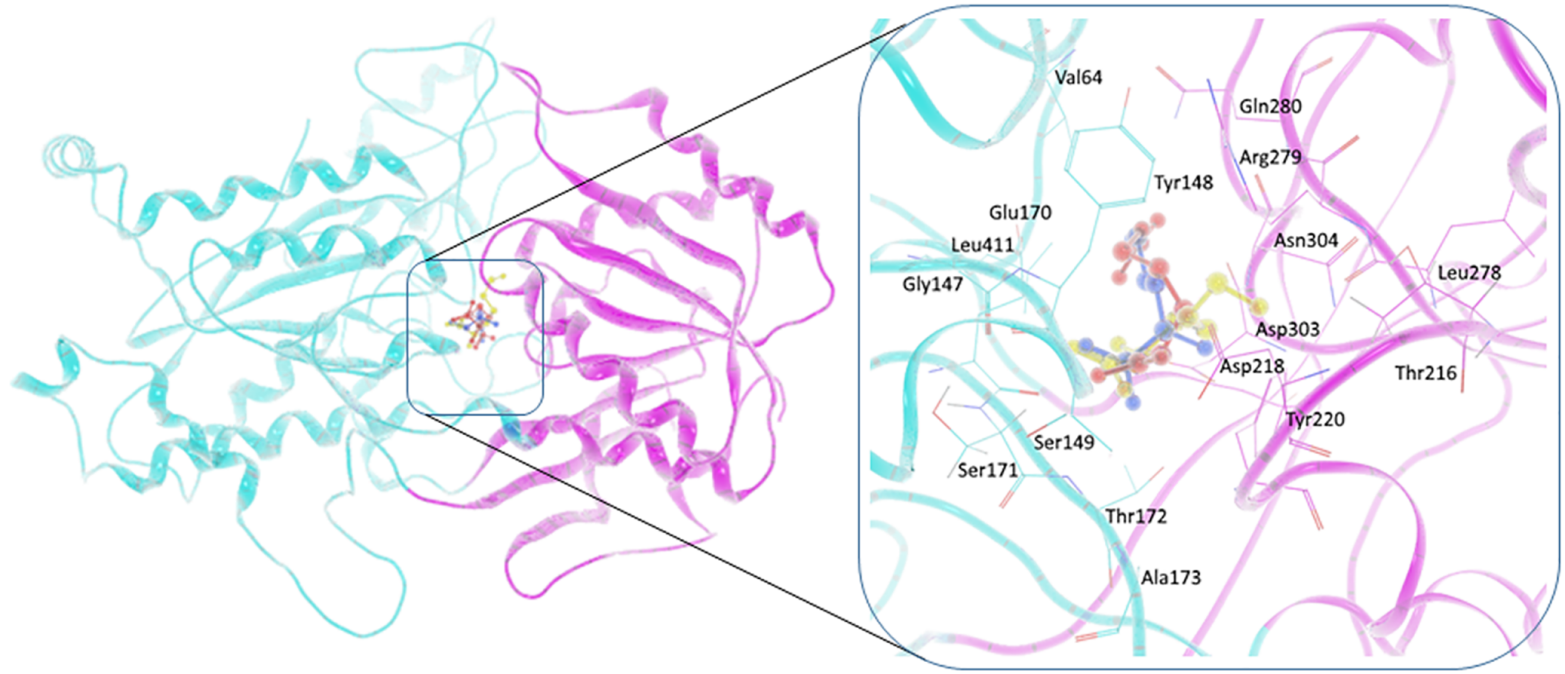
Docking of amino acids to GPRC6A VFT homology model. Left, Orthosteric site in the VFT domain, Arg is shown in red ball-stick representation, Lys in yellow and ornithine in blue. Right, Consensus binding site residues surrounding the amino acid in the binding pocket.

The GPRC6A VFT homology model is shown in Fig 1. The GPRC6A sequence exhibits similarities of 42.4% and 28.5% to the mGluR-3 and GABAR-1, respectively (sequences 4 and 6 in S1A Fig). The mGluR-3 structure was selected as the main template for the homology modeling calculations and the corresponding structural models selected for docking studies. The highest scoring structural model is shown in S1B Fig. Nineteen possible allosteric binding site residues in the TM domain have been identified in our previous work [21], corresponding to the common allosteric site for family C GPCRs. Interestingly, the binding site in the VFT domain was found to be present at the hinge between the two subdomains, suggesting a possible hinge-bending binding mechanism. A total of 17 orthosteric binding-site residues were identified for basic amino acid ligands for GPRC6A in the VFT domain; these are listed in Table 1 and S2 Fig, and shown in Fig 1. Out of these binding pocket residues, conserved Ser149 and Thr172 have been found experimentally to be important for binding amino acids [46]. Non-conserved Glu170 and conserved Asp303 are other important residues predicted to interact with all three amino acids.

**Table 1:** Orthosteric binding-site residues in the VFT for basic amino acid ligands for GPRC6A.

Docked Amino acids	Orthosteric binding-site residues
L-Arg	Val64, Gly147, Tyr148, Ser149, Glu170, Ser171, Thr172, Asp218, Tyr220, Gln280, Asp303, Asn304, Leu411
L-Lys	Gly147, Tyr148, Ser149, Glu170, Ser171, Thr172, Thr216, Asp218, Tyr220, Leu278, Arg279, Asp303, Asn304
L-Ornithine	Val64, Gly147, Tyr148, Ser149, Glu170, Ser171, Thr172, Ala173, Tyr220, Gln280, Asp303, Asn304, Leu411
Consensus site (residues interacting with at least one amino acid)	Val64, Gly147, Tyr148, Ser149, Glu170, Ser171, Thr172, Ala173, Thr216, Asp218, Tyr220, Leu278, Arg279, Gln280, Asp303, Asn304, Leu411

### Dynamics of GPRC6A TM and VFT domains

**Fig 2:**
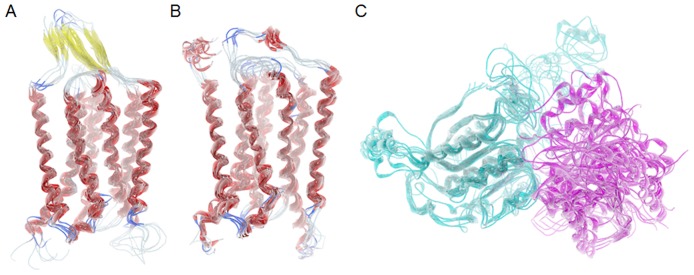
The molecular structures from GPRC6A homology modeling. Representative structures from the 100ns MD simulations of the (A) TM model structure based on mGluR-1 template structure, (B) TM model structure based on mGluR-5 template structure, and (C) VFT model structure based on mGluR-3 template structure.

**Fig 3:**
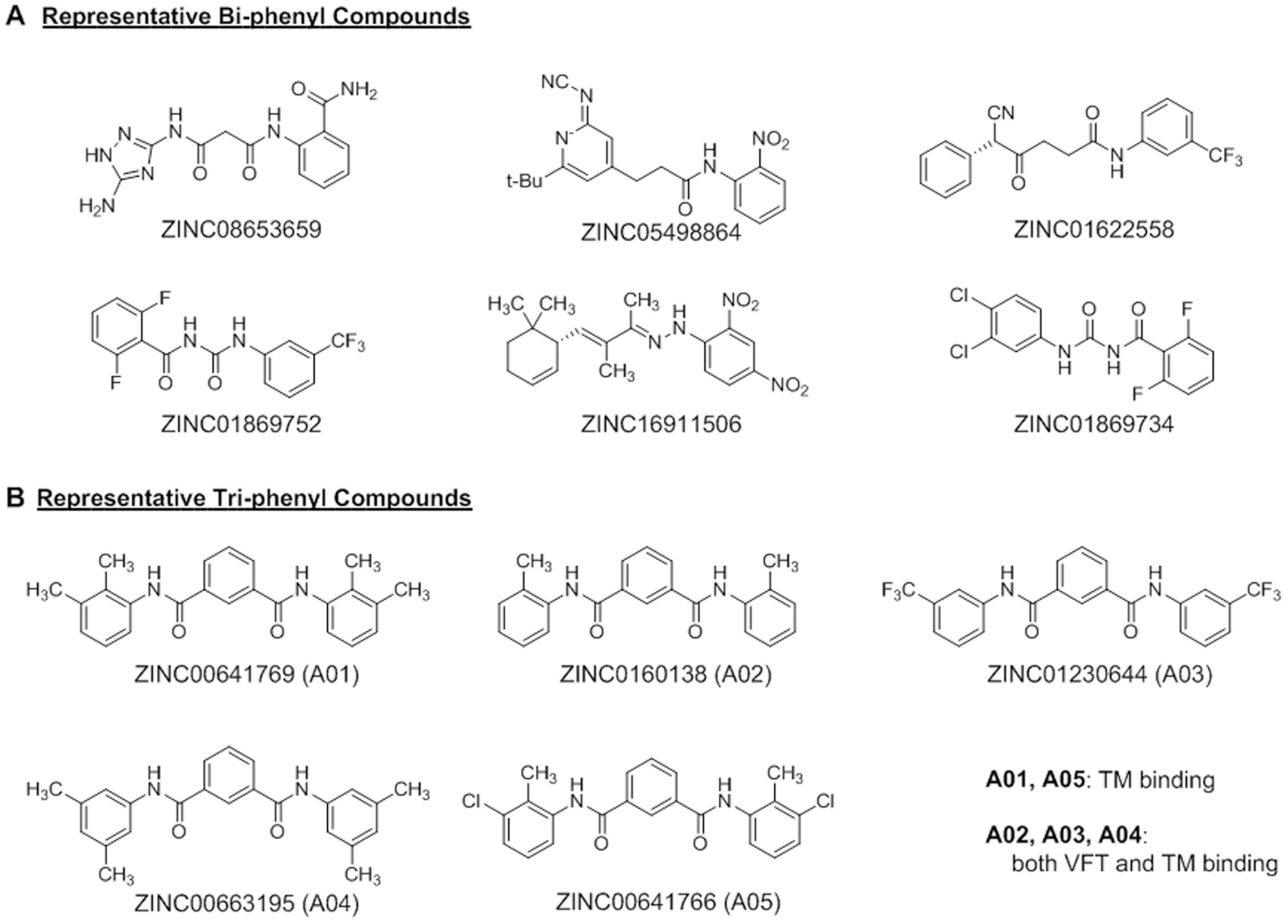
The molecular structures of the compounds with high binding score for GPRC6A from computational screening. The molecular structures of six bi-phenyl compounds with calculated high binding score for the VFT of GPRC6A (A), and five tri-phenyl compounds with calculated high binding score for TM of GPRC6A (B) from 90,000 molecule computational screening based on mGluR-3 template. The compounds, A01 and A05 are predicted to bind to TM domain of GPRC6A. The compounds, A02, A03 and A04 are predicted to bind to both VTM and TM of GPRC6A.

We assessed the dynamics of the two domains of GPRC6A through MD simulations. A clustering of 27 allosteric binding site residues in TM domain was used to identify 12 representative structures, 6 each from the two 100ns trajectories of the 2 model structures (including the 2 starting homology models), shown in Fig 2A and 2B. Clustering of 17 orthosteric binding site residues in the VFT domain was used to identify 6 representative structures from the 100ns trajectory (including 1 starting homology model), shown in Fig 3. The VFT domain was found to open during the course of the MD, increasing the size of the binding pocket in the later snapshots in the MD identified through clustering. Stabilizing the open conformation of the VFT domain may provide an alternate route for developing antagonists, where these compounds prevent the VFT to form the close conformation necessary for binding amino acids and the transfer of signals to the TM domain.

### Virtual screening and Shortlisting compounds

The 18 selected structures of the VFT and TM domains of GPRC6A were each screened against a library of 89350 compounds. S1 and S2 Tables show compounds with highest binding affinities, as predicted by Vina, for the TM and VFT domains, respectively, derived from the starting homology models and MD snapshots. The compounds ranked in top-5000 (based on predicted binding energies) showing highest snapshot-count for the TM and VFT domains are shown in S3 and S4 Tables, respectively. The compounds found to bind to both TM and VFT domains (49 compounds) are listed in S5 Table. Thirteen of the 32 compounds predicted to bind with the highest affinities to the VFT contained bi-phenyl moieties (Fig 3A), whereas compounds predicted to bind to both the VFT and TM domains belonged predominantly to a class of tri-phenyl dipeptides (Fig 3B), (all with binding free energies o<-12.6kcal/mol).

### Functional analysis of tri-phenyl compounds *in vitro*

We previously tested selective androgen modulators (SARM), which are structurally similar bi-phenyl compounds. Since these bi-phenyl activate GPRC6A as well as the androgen receptor in heterologous reporter assay [21], we did not evaluate the additional bi-phenyl compounds that we identified in the current screen. Rather, we focused on the newly identified tri-phenyl compounds.

**Fig 4:**
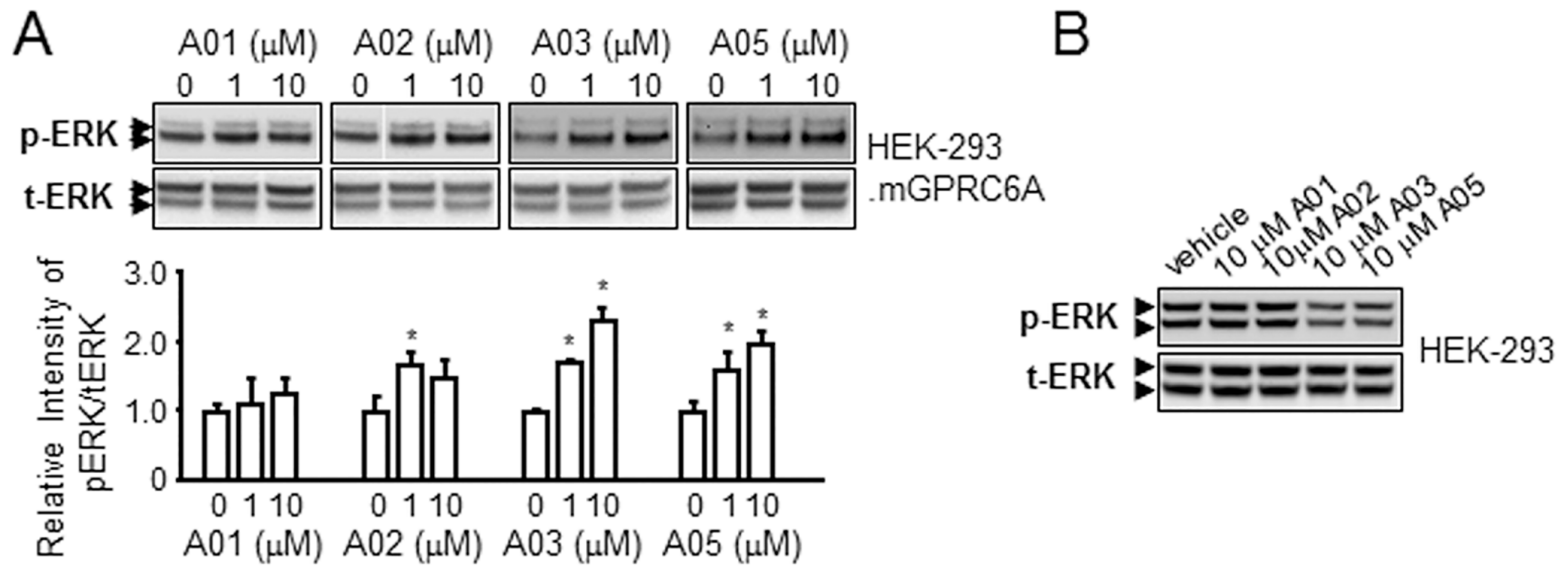
The results for novel tri-phenyl compounds. The tri-phenyl compounds stimulated ERK activation in HEK-293 cells transfected with GPRC6A (A) and untransfected HEK-293 controls (B).

We obtained the five tri-phenyl compounds shown in Fig 3B to experimentally test their ability to stimulate GPRC6A mediated ERK phosphorylation in HEK-293 cells transfected with mouse GPRC6A cDNA construct. Compound A04 is insoluble in water, DMSO or alcohol and could not be tested. The novel compound, A03 showed higher activity to stimulate ERK phosphorylation in HEK-293 cells transfected with mGPRC6A cDNA compared to A01, A02 and A05 (Fig 4A). These compounds did not activate ERK in HEK-293 cells not transfected with GPRC6A (Fig 4B).

**Fig 5:**
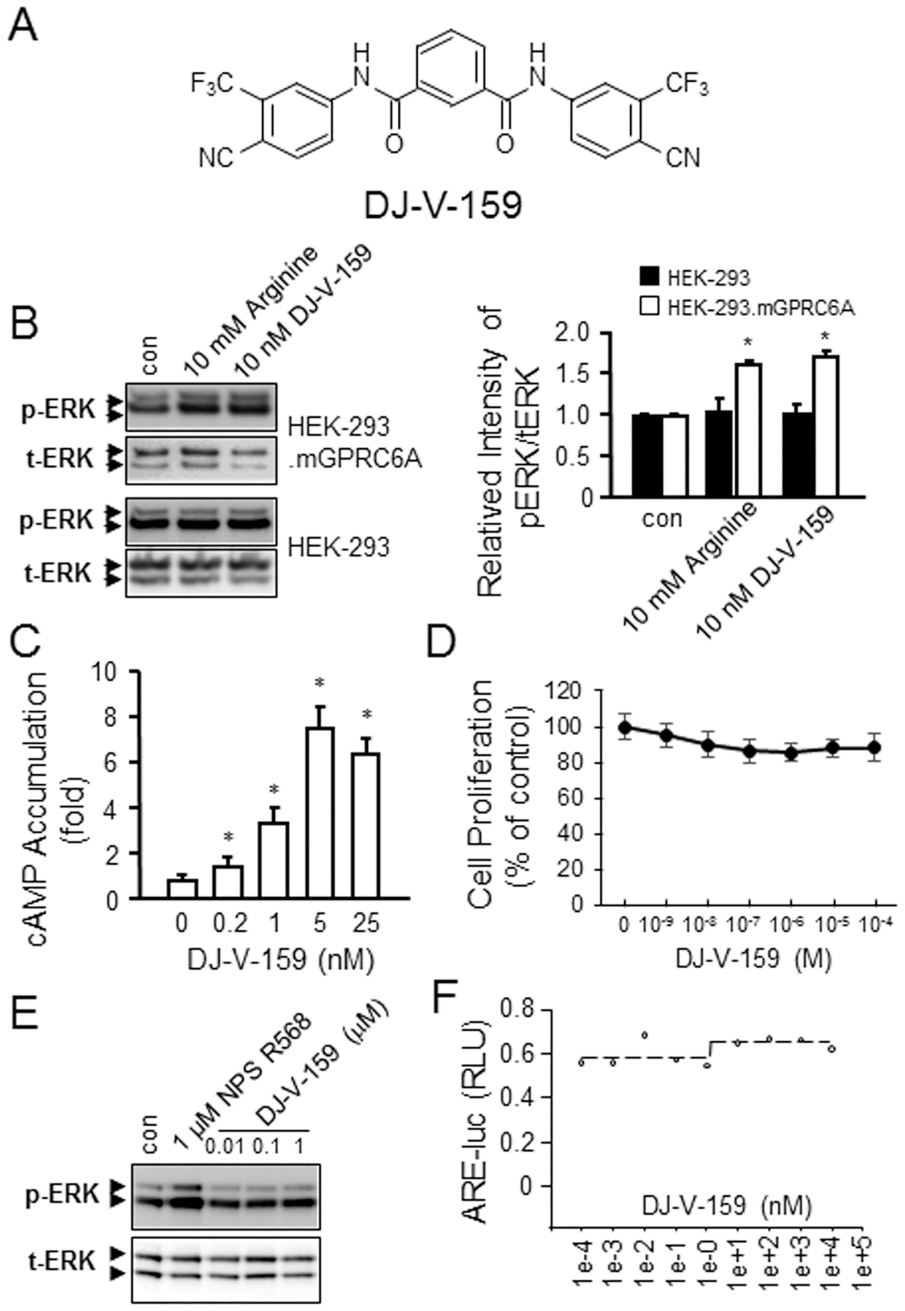
Function analysis of novel compound, DJ-V-159 *in vitro*. (A) Structure of DJ-V-159. DJ-J-159 [*N*^1^,*N*^3^-bis(4-Cyano-3-(trifluoromethyl)phenyl)isophthalamide] derived from A03. DJ-V-159 stimulated GPRC6A-mediated ERK phosphorylation (B) and cAMP accumulation (C). (D) The cytotoxicity assay of DJ-V-159 in HEK-293 cells. The selectivity assay in HEK-293 cells transfected with human CasR (E) or human AR (F), respectively.

We synthesized a novel tri-phenol compound, DJ-V-159 to test the biological response of the tri-phenyl scaffold *in vitro* (Fig 5A). DJ-V-159 was derived from A03 by chemical modifications of substituted-aniline and isophthaloyl dichloride. We confirmed that DJ-V-159 activates ERK in HEK-293 transfected with GPRC6A but not in non-transfected HEK-293 cells, with potency similar to L-Arg (Fig 5B). In addition, DJ-V-159 dose-dependently stimulated cAMP production in GPRC6A expressing HEK-293 cells, achieving a response a 0.2 nM concentrations in the media (Fig 5C). No activation was observed in non-transfected HEK-293 cells (Fig 5B). We also checked the cytotoxicity of DJ-V-159, in this assay, we treated HEK-293 cells with DJ-V-159 at 0, 10^−9^, 10^−8^, 10^−7^, 10^−6^, 10^−5^ and 10^−4^ M concentrations for 72 hours. Following 72 hours of treatment, relative viable cell number was determined. No cytotoxicity of DJ-V-159 in HEK-293 cells was observed after 3-day culture (Fig 5D). DJ-V-159 was also selective, since it did not stimulate ERK phosphorylation in HEK-293 cells transfected with the closely-related calcium sensing receptor (CasR) (Fig 5E). In contrast to the bi-phenyl compound DJ-I-267 that shows sterospecific activation of AR [21], we found that DJ-V-159 did not activate AR signaling in HEK-293 cells, indicating that the middle phenol increases GPRC6A specificity (Fig 5F).

**Fig 6:**
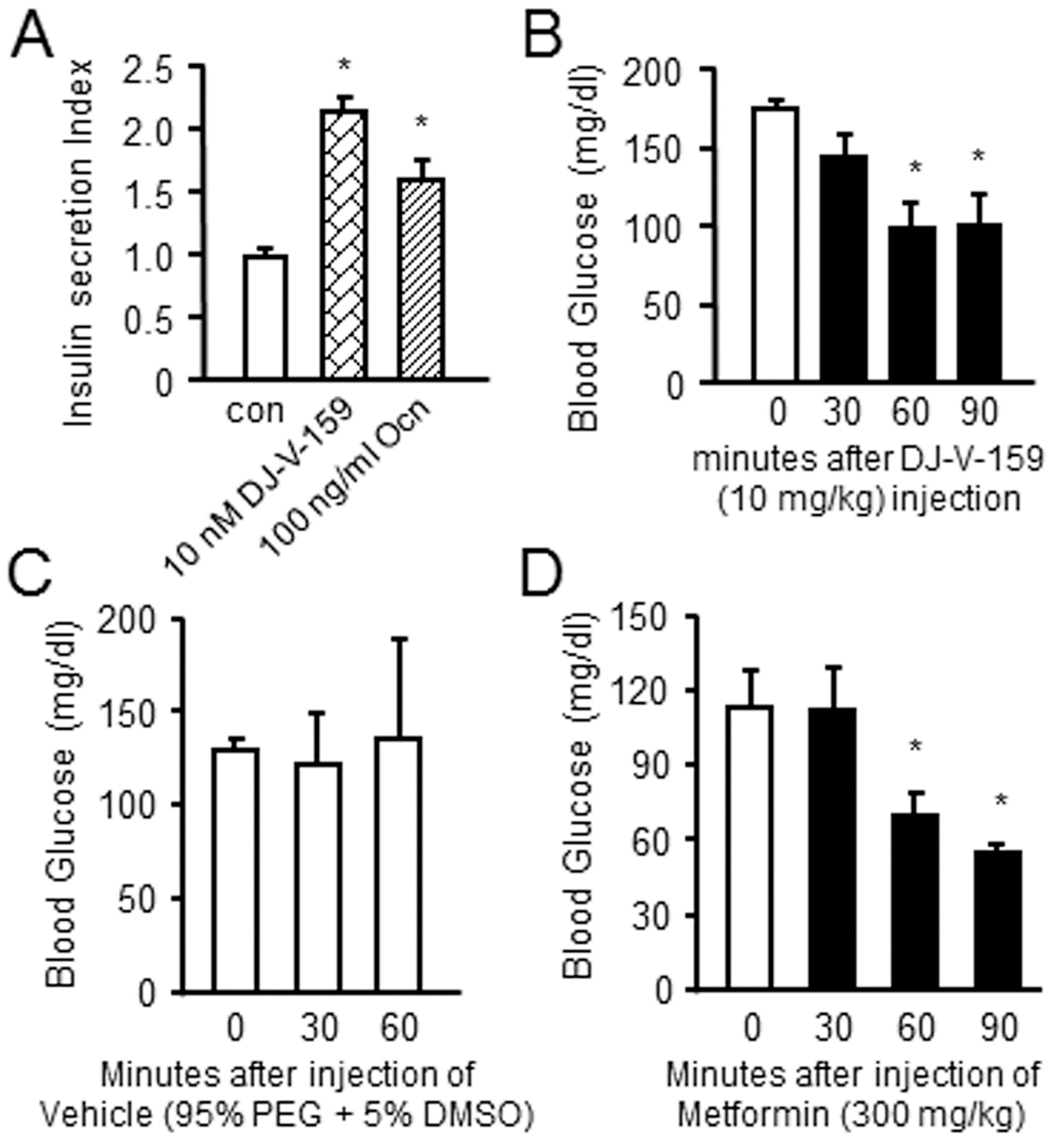
*In vivo* effects of DJ-V-159. (A) The effects of DJ-V-159 on GPRC6A-mediated regulation of insulin secretion in mouse β-cells, MIN-6. (B and C) The biological activity of DJ-V-159, after 30 minutes injected 10 mg/kg DJ-V-159 (B) or vehicle (95% PEG + 5% DMSA) (C) by IP to wild type mouse. The blood glucose were measured, DJ-V-159 significantly decreased blood glucose level compared to vehicle treated in wild type mice. (D) Effects of Metformin to lower blood glucose in wild type mice after 30 minutes IP injected 300 mg/kg Metformin, the blood glucose was measured.

Next, to investigate the effects of DJ-V-159 on GPRC6A-mediated regulation of insulin secretion, we performed insulin secretion studies in mouse β-cells, MIN-6. We found that DJ-V-159 stimulated insulin secretion in mouse beta-cell MIN-6 cells. The DJ-V-159 increased insulin stimulation index (SI) in MIN-6 cells similar to the effects of Ocn, known ligand of GPRC6A (Fig 6A). We also tested the biological response of the tri-phenyl scaffold *in vivo* (Fig 6B). We found that DJ-V-159 at the dose of 10 mg/kg reduced blood glucose levels in wild-type mice at 60 and 90 minutes after intraperitoneal administration of 10 mg/kg (Fig 6B), whereas the vehicle (95% PEG + 5% DMSO) had no effect on blood glucose (Fig 6C). DJ-V-159 reduced blood glucose levels in wild-type mice by 43.6% and 41.9% at 60 and 90 minutes, respectively, after intraperitoneal [47] administration of 10 mg/kg (Fig 4B). Finally, we compared the response of DJ-V-159 in reduction of blood glucose to metformin. Doses of metformin of 300 mg/kg resulted in similar reductions in blood glucose of 45.5% and 54.2% at 60 and 90 minutes, respectively, after IP administration, but using a dose of 300 mg/kg, that is 30-fold higher than that required for DJ-V-159 at 10 mg/kg to observe similar glucose reductions (Fig 4D).

The mice tolerated this short-term exposure to DJ-V-159 without any overt side-effects. DJ-V-159, however, is almost in or on the boundary of the Lipinski’s Rule of Five, with the following properties: molecular weight is 502.37 (recommended below 500); calculated Log P is 5.55 (recommended −0.4 to +5.6); H-donor and acceptor: 4 or 6 (recommended less than 5); and number of atoms 48 (recommended 20 to 70).

## Discussion

In this study, we have pursued a target-based strategy to identify lead compounds that activate GPRC6A. Using homology modeling, MD simulation, and virtual high-throughput screening to guide experiments, we identified bi-phenyl compounds that are predicted to bind to the VFT, and tri-phenyl compounds predicted that are predicted to bind to either the 7-TM or to both the VFT and 7-TM domains (S3 and S4 Figs). Experimental testing of 4 of the newly discovered tri-phenyl compounds, found that A03 and A05 activated GPRC6A-dependent ERK phosphorylation and cAMP accumulation in a dose-dependent fashion, with an EC50 and % max of 1.11 nM, and 80%. The identification of bi-phenyl and tri-phenyl compounds that activate GPRC6A validates our virtual high-throughput screening approach that identifies molecular interactions of chemical probes with the GPRC6A as a drug discovery tool.

A03 was chemically modified to create DJ-V-159 for further testing. DJ-V-159 has the molecular formula N1, N3-bis(4-cyano-3-(trifluoromethyl)phenyl)isophthalamide and a molecular weight of 502.37 (S5 and S6 Figs). The scaffold exemplified by DJ-V-159 is an improvement over the natural ligands for GPRC6A. The responses to DJ-V-159 are similar to reported effects of GPRC6A’s natural ligand, Ocn, which stimulates insulin secretion and lowers blood glucose concentrations at the doses of 1 to 3 μg/kg administered systemically to mice [22]. Ocn has not yet been developed as a peptide therapeutic, but will likely be limited due to poor chemical and physical stability, short half-life, and thus a need for systemic administration. Indeed, Ocn treatment at the dose of 1 and 3 μg/kg is required to stimulate insulin levels and insulin gene expression in mice. DJ-V-159 is more specific than other natural GPRC6A ligands, such as T and L-Arg, which have off-target effects to activate the androgen receptor and directly affect cellular metabolism, respectively. Moreover, L-Arg requires doses in excess of 2 g/kg to achieve a transient metabolic response [21, 27, 31].

Although DJ-V-159 marginally conforms to the Rule of Five that predicts drug-like properties, DJ-V-159 is a potential lead compound because it: i) exhibits a predicted strong binding score to the receptor, ii) is representative of a chemical scaffold that performs well in the virtual screening calculations, and iii) is chemically different from the bi-phenyl compounds, like DJ-I-267, that activate both GPRC6A and the androgen receptor (AR), hence adding a strong potential for specificity as well as potency [21]. Most importantly, DJ-V-159 dose dependently activated GPRC6A in screening assays. Activation of GPRC6A by DJ-V-159 occurs a 0.2 nM concentrations. In addition, DJ-V-159 stimulated insulin secretion in mouse β-cells and lowered blood glucose when administered systemically to wild-type mice. The response to DJ-V-159 in mice was similar in magnitude to metformin, which is a first-line therapy for T2D that targets hepatic glucose production [48]. Thus, while DJ-V-159 on- and off-target liabilities, pharmacokinetics and ADMET studies need to be defined, the available data indicate that DJ-V-159 represents a favorable chemical probe which could be used as a starting point to develop a safe and effect drug to lower plasma glucose, increases insulin sensitivity, and reduces fat mass in T2D [49], through activation of GPRC6A. Even if DJ-V-159 proves to have liabilities that prevent its progression as a drug candidate, modifications of the scaffold offer the potential to develop an optimal safe and effective ligand for GPRC6A.

In conclusion, we have identified GPRC6A as a new therapeutic target for treating T2D and identified small molecules that selectively activate GPRC6A leading to stimulation of insulin secretion *in vitro* and lowering of serum glucose in mice. These preliminary findings set the stage for lead optimization of a chemical series of GPRC6A agonists to optimize potency, selectivity, and biological activity to fulfill the criteria for a potentially new therapeutic.

## Supporting information

S1 Fig GPRC6A VFT homology modelling. (A) Sequence similarity scores between 16 sequences after MSA. Sequence 1: GPRC6A, Sequences 2–9: family C GPCR’s, Sequences 10–16: family A GPCR’s. mGluR-3 (sequence-4) taken as main templates for VFT domain modelling. (B) GPRC6A VFT homology model based on the mGlu-3 receptor structure.
(DOCX)

S2 Fig Docking of amino acids to VFT domain. Residues in binding pocket surrounding Arginine (A), Lysine (B), and Ornithine (C).
(DOCX)

S3 Fig Docking of compounds A01-A05 to TM. Red: A01, green: A02, blue: A03, yellow: A04, and pink: A05.
(DOCX)

S4 Fig Docking of compounds A03 and A04 to VFT of GPRC6A. Yellow: A03 and green: A04.
(DOCX)

S5 Fig Docking of compounds DJ-V-159 to the TM of GPRC6A. Yellow: DJ-V-159.
(DOCX)

S6 Fig The possible binding mode of DJ-V-159 in the VFT of GPRC6A conformations. Yellow: DJ-V-159.
(DOCX)

S1 Table TM domain compounds with highest binding affinities. (DOCX)

S2 Table VFT domain compounds with highest binding affinities. (DOCX)

S3 Table TM domain compounds binding to maximum number of snapshots. (DOCX)

S4 Table VFT domain compounds binding to maximum number of snapshots. (DOCX)

S5 Table Compounds from above lists found to be binding in both TM and VFT domains. (DOCX)

## Abbreviations

Cur: Curcumin
GLP-1: Glucagon-like peptide-1
GPCR: G protein coupled receptor
GPRC6A: G protein coupled receptor family C group 6 member A
IL-6: Interleukin 6
MD: molecular dynamics
MetS: metabolic syndrome
Ocn: Osteocalcin
T: Testosterone
T2D: type 2 diabetes mellitus
TM: transmembrane
VFT: venus fly trap
