# Supplementary material for: Computationally identified novel agonists for GPRC6A
Source: PLoS One. 2018 Apr 23;13(4):e0195980. doi: 10.1371/journal.pone.0195980 (PMC5912754; doi:10.1371/journal.pone.0195980)
Supplement: S1 Fig — (A) Sequence similarity scores between 16 sequences after MSA. Sequence 1: GPRC6A, Sequences 2–9: family C GPCR’s, Sequences 10–16: family A GPCR’s. mGluR-3 (sequence-4) taken as main templates for VFT domain modelling. (B) GPRC6A VFT homology model based on the mGlu-3 receptor structure. (DOCX) [file pone.0195980.s001.docx]

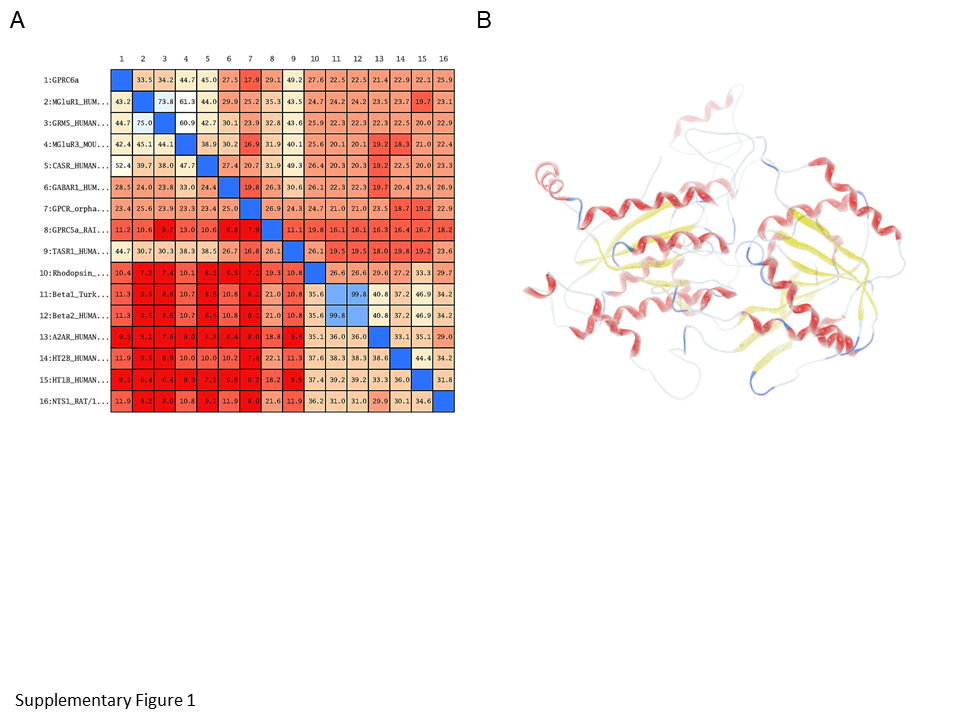


**S1 Fig. GPRC6A VFT Homology Modelling.** (A) Sequence similarity scores between 16 sequences after MSA. Sequence 1: GPRC6A, Sequences 2-9: family C GPCR's, Sequences 10-16: family A GPCR's. mGluR-3 (sequence-4) taken as main templates for VFT domain modelling. (B) GPRC6A VFT homology model based on the mGlu-3 receptor structure.
