# Supplementary material for: Computationally identified novel agonists for GPRC6A
Source: PLoS One. 2018 Apr 23;13(4):e0195980. doi: 10.1371/journal.pone.0195980 (PMC5912754; doi:10.1371/journal.pone.0195980)
Supplement: S2 Fig — Residues in binding pocket surrounding Arginine (A), Lysine (B), and Ornithine (C). (DOCX) [file pone.0195980.s002.docx]

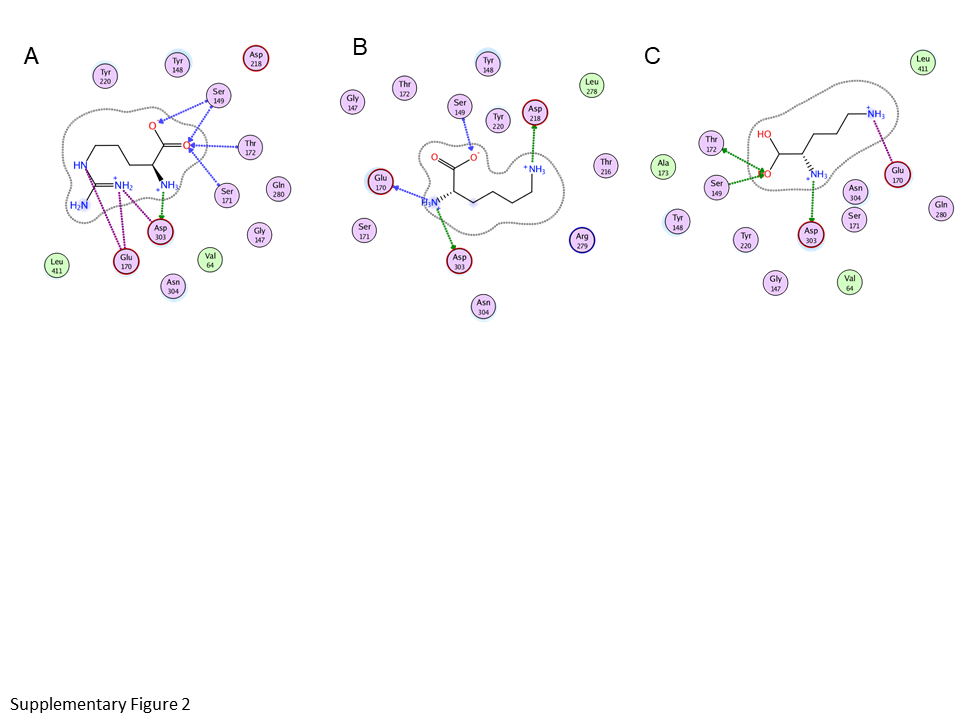


**S2 Fig. Docking of Amino Acids to VFT domain.** Residues in binding pocket surrounding Arginine (A), Lysine (B), and Ornithine (C).
