# Supplementary figures and images for: Computationally identified novel agonists for GPRC6A
Source: PLoS One. 2018 Apr 23;13(4):e0195980. doi: 10.1371/journal.pone.0195980 (PMC5912754; doi:10.1371/journal.pone.0195980)

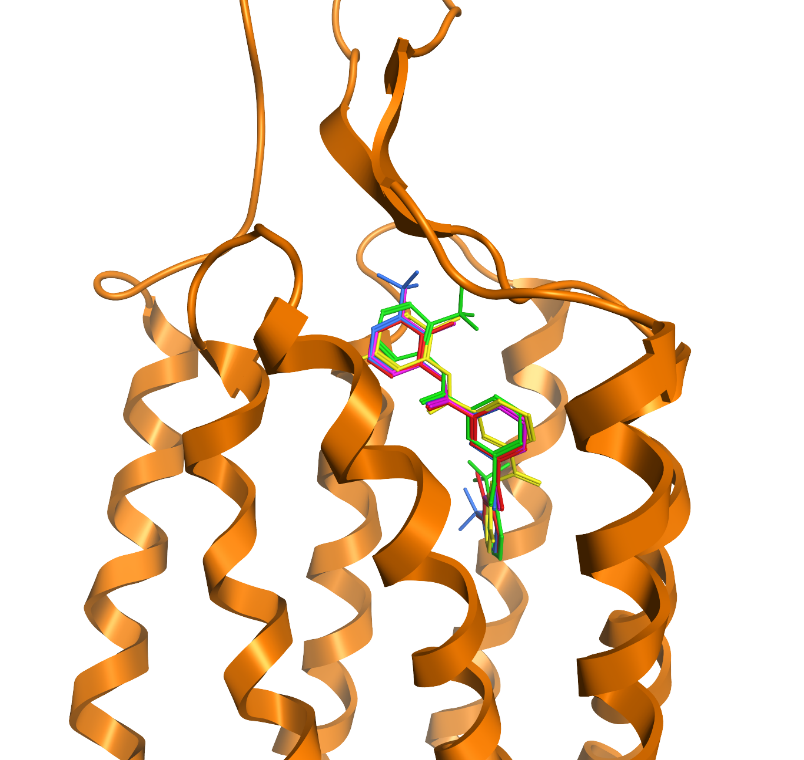

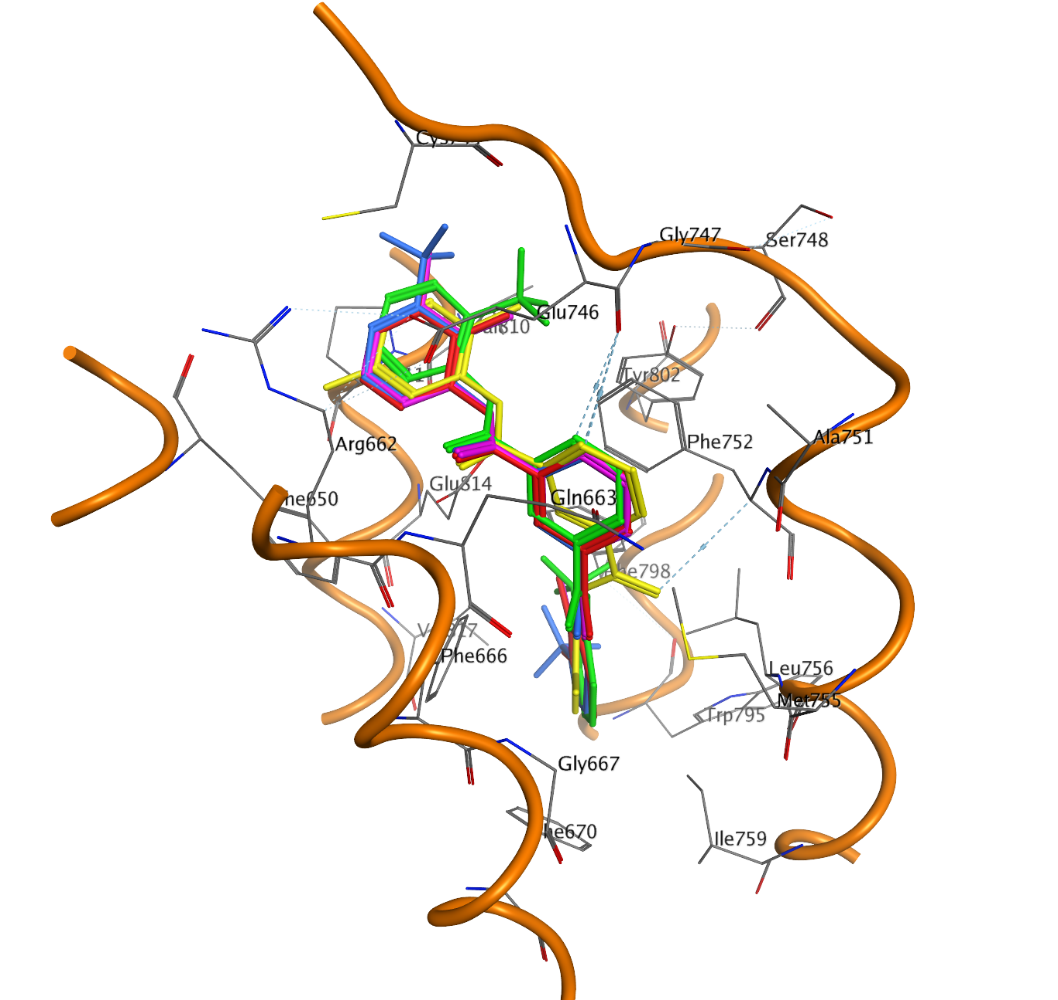


**S3 Fig. Docking of compounds A01-A05 to TM.** Red: A01, green: A02, blue: A03, yellow: A04, and pink: A05.

Supplement: S3 Fig — Red: A01, green: A02, blue: A03, yellow: A04, and pink: A05. (DOCX) [file pone.0195980.s003.docx]

**
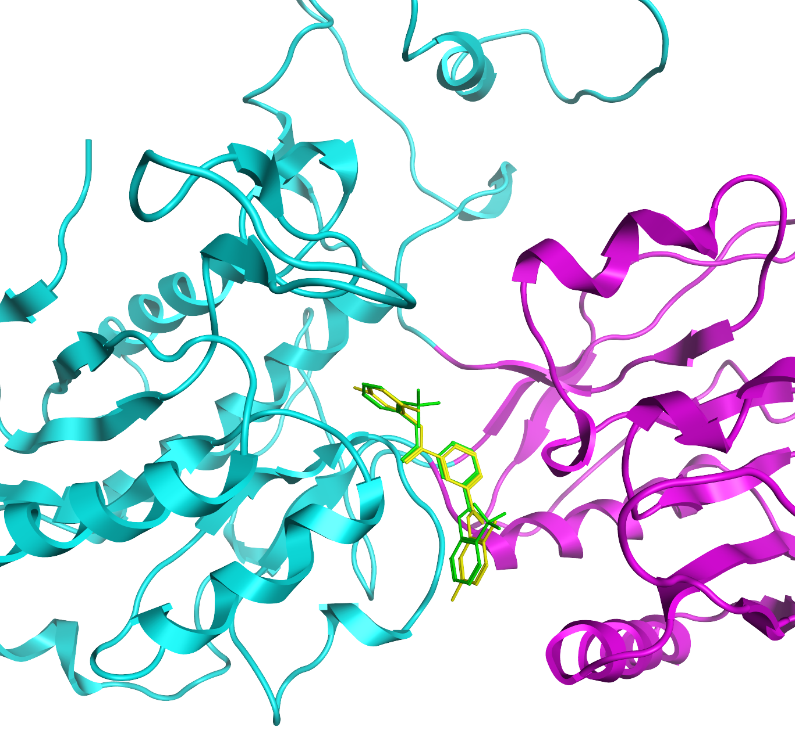

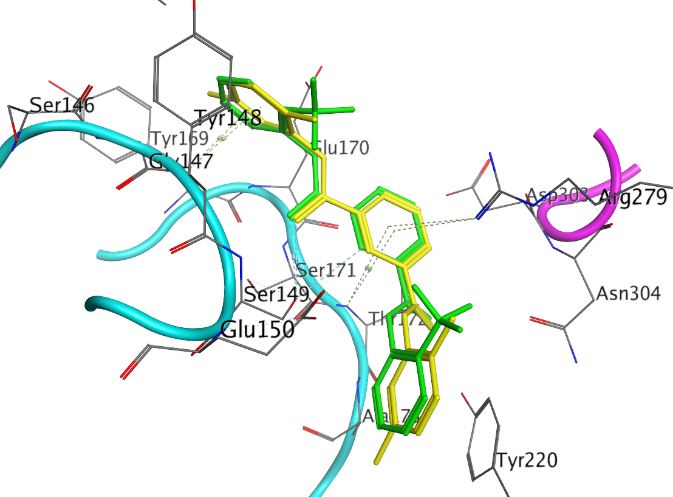
**

**S4 Fig. Docking of compounds A03 and A04 to VFT of GPRC6A.** Yellow: A03 and green: A04.

Supplement: S4 Fig — Yellow: A03 and green: A04. (DOCX) [file pone.0195980.s004.docx]

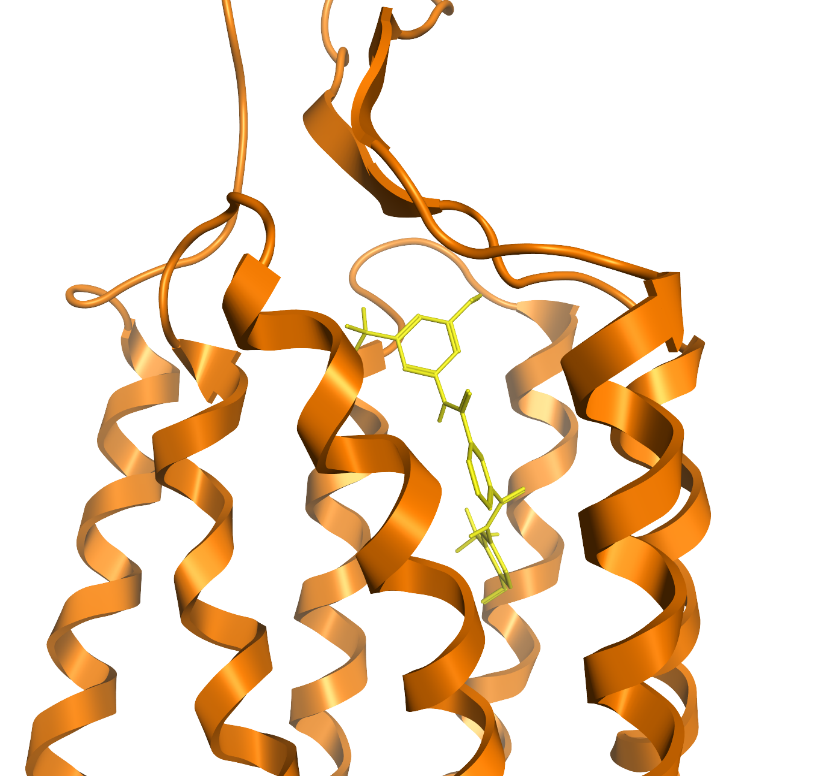

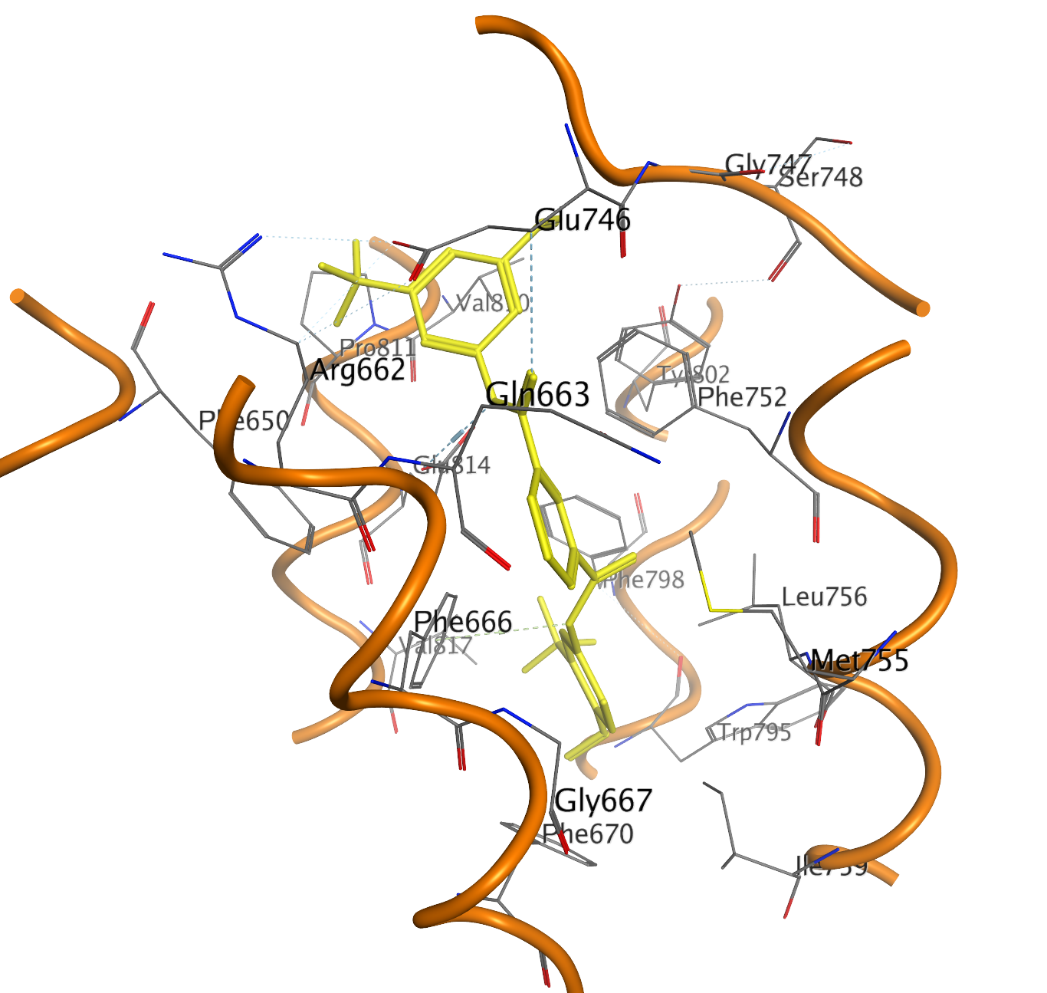


**S5 Fig. Docking of compounds DJ-V-159 to the TM of GPRC6A.** Yellow: DJ-V-159.

Supplement: S5 Fig — Yellow: DJ-V-159. (DOCX) [file pone.0195980.s005.docx]

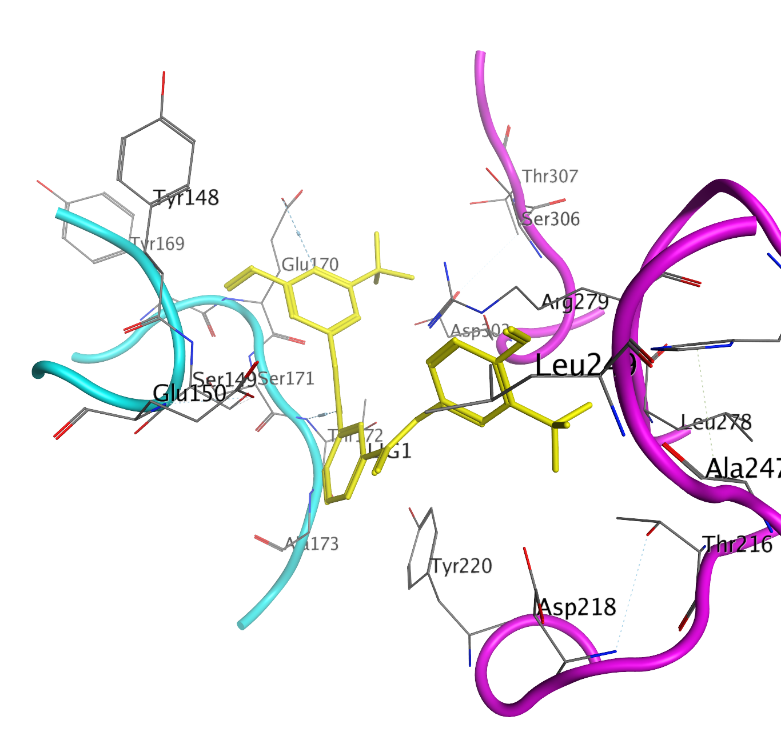

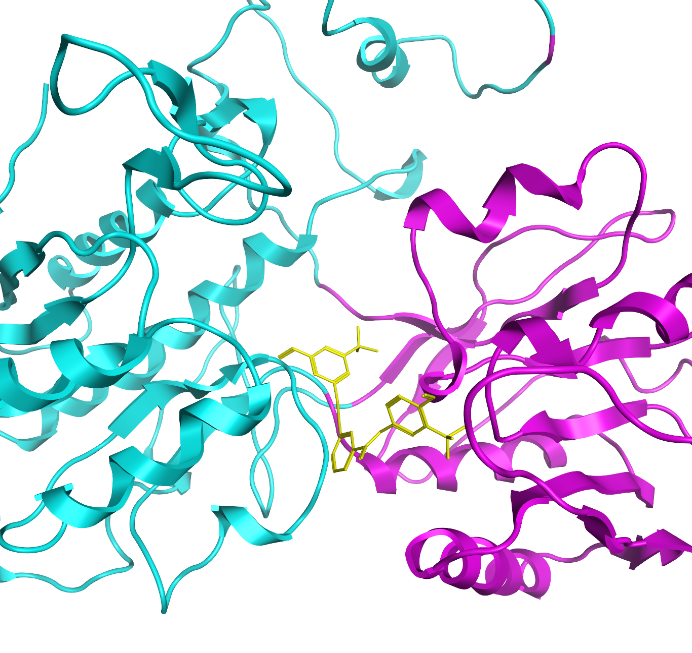


**S6 Fig. The possible binding mode of DJ-V-159 in the VFT of GPRC6A conformations.** Yellow: DJ-V-159.

Supplement: S6 Fig — Yellow: DJ-V-159. (DOCX) [file pone.0195980.s006.docx]

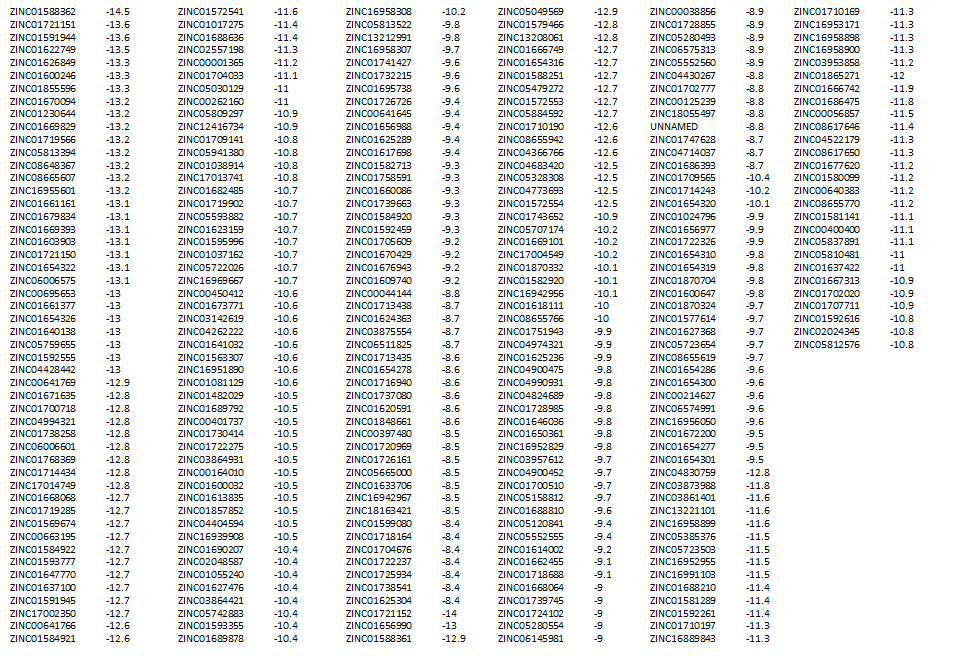


**S1 Table. TM domain compounds with highest binding affinities.**

Supplement: S1 Table — (DOCX) [file pone.0195980.s007.docx]

**
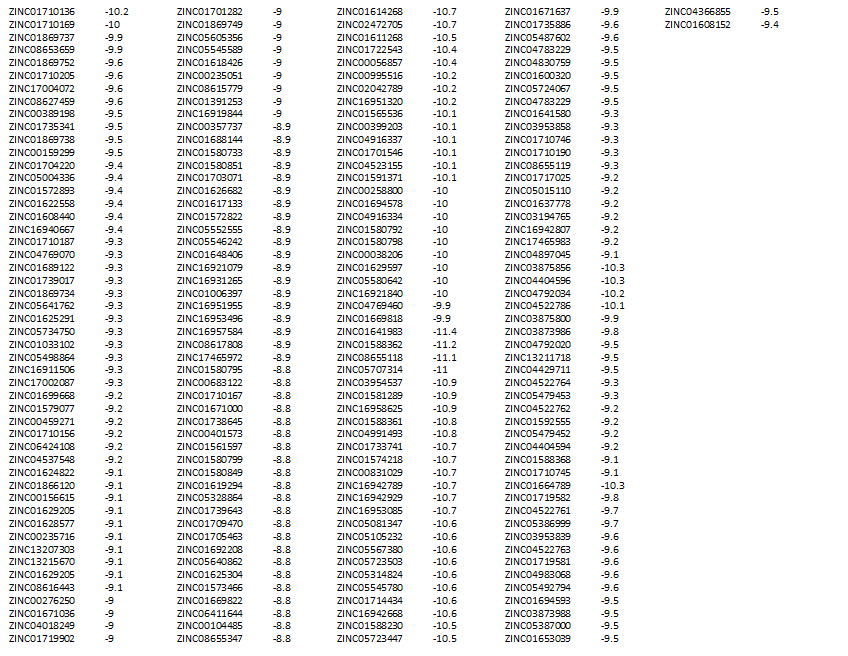
**

**S2 Table. VFT domain compounds with highest binding affinities.**

Supplement: S2 Table — (DOCX) [file pone.0195980.s008.docx]

**
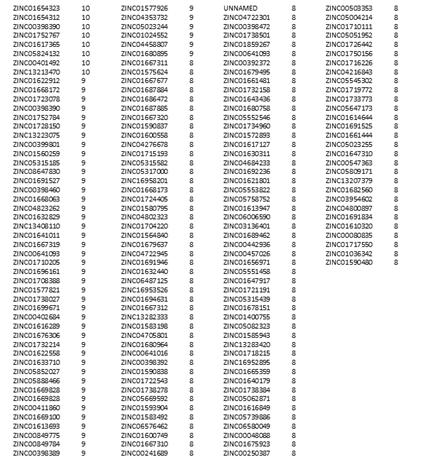
**

**S3 Table. TM domain compounds binding to maximum number of snapshots.**

Supplement: S3 Table — (DOCX) [file pone.0195980.s009.docx]

**
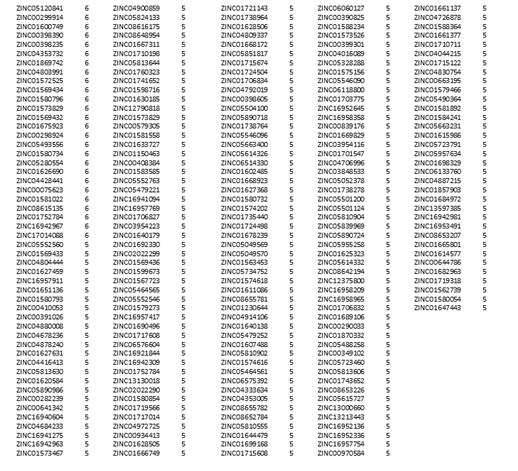
**

**S4 Table. VFT domain compounds binding to maximum number of snapshots.**

Supplement: S4 Table — (DOCX) [file pone.0195980.s010.docx]

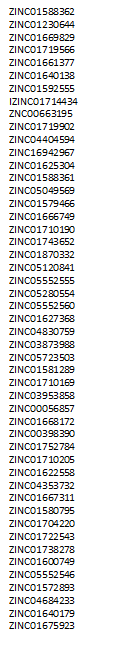


**S5 Table. Compounds from above lists found to be binding in both TM and VFT domains.**

Supplement: S5 Table — (DOCX) [file pone.0195980.s011.docx]
